# The Involvement of PDE4 in the Protective Effects of Melatonin on Cigarette-Smoke-Induced Chronic Obstructive Pulmonary Disease

**DOI:** 10.3390/molecules26216588

**Published:** 2021-10-30

**Authors:** Je-Oh Lim, Woong-Il Kim, Se-Jin Lee, So-Won Pak, Young-Kwon Cho, Jong-Choon Kim, Joong-Sun Kim, In-Sik Shin

**Affiliations:** 1 BK21 FOUR Program, College of Veterinary Medicine, Chonnam National University, 77 Yongbong-ro, Buk-gu, Gwangju 61186, Korea; 166634@jnu.ac.kr (J.-O.L.); 208521@jnu.ac.kr (W.-I.K.); 218729@jnu.ac.kr (S.-J.L.); 208514@jnu.ac.kr (S.-W.P.); toxkim@jnu.ac.kr (J.-C.K.); centraline@jnu.ac.kr (J.-S.K.); 2 College of Health Science, Cheongju University, Chungbuk 28503, Korea; petmen@hanmail.net

**Keywords:** melatonin, chronic obstructive pulmonary disease, cigarette smoke, phosphodiesterase 4, MMP-9

## Abstract

Chronic obstructive pulmonary disease (COPD) is a significant disease threatening human health. Currently, roflumilast, a phosphodiesterase (PDE)4 inhibitor, is recommended as a therapeutic agent for COPD. In this study, we investigated the therapeutic effects of melatonin against COPD, focusing on determining whether it is a PDE4 inhibitor via in vivo and in vitro experiment using cigarette smoke (CS) and cigarette smoke condensate (CSC), respectively. In the in vivo experiments, melatonin treatment reduced inflammatory responses, including inflammatory cell counts. Melatonin treatment also suppressed the CS-exposure-induced upregulation of cytokine and matrix metalloproteinase (MMP)-9, reduced the PDE4B expression, and elevated cAMP levels. In addition, these effects were synergistic, as melatonin and roflumilast cotreatment eventually ameliorated the CS-exposure-induced worsening of lung function. In the CSC-stimulated NCI-H292 cells, melatonin inhibited elevation in the levels of inflammatory cytokines, MMP-9, and PDE4, and elevated cAMP levels. Furthermore, melatonin and roflumilast cotreatment was more effective on inflammatory responses than only melatonin or roflumilast treatment. Our results indicate that melatonin relieves inflammatory response and loss of lung function in COPD, which is associated with decreased PDE4 expression. Therefore, we suggest that melatonin is a putative candidate for the treatment of COPD.

## 1. Introduction

The prevalence and mortality associated with chronic obstructive pulmonary disease (COPD) continues to increase worldwide. It is thus regarded as an important disease threatening human health [1]. COPD is characterized by pulmonary inflammation, bronchoconstriction, and mucus production, which eventually induce airflow restrictions due to loss of elastic recoil of the pulmonary tract [2]. Cigarette smoke (CS) is regarded as a crucial cause for the development of COPD, and it generates various stimuli, such as proinflammatory mediators and oxidative stress, resulting in pulmonary inflammation [3]. Continuous smoking increases the incidence of COPD via the elevation of inflammatory responses and oxidative damage. Therefore, inhibition of the inflammation and oxidative damage induced by CS is considered an important strategy for the effective control of COPD [4].

Loss of lung function caused by CS is closely associated with cyclic adenosine monophosphate (cAMP) and cyclic guanosine monophosphate (cGMP) [5]. Continuous smoking increases the expression of phosphodiesterase (PDE) in inflammatory cells, including neutrophils and macrophages, which promotes the conversion of cAMP to AMP, resulting in the decline of normal lung function [6,7,8]. Therefore, the inhibition of PDE expression ameliorates the CS-induced worsening of lung function via the elevation of cAMP levels. Based on these observations, the use of PDE inhibitors as therapeutic drugs has been recommended for COPD treatment [9]. Among PDE inhibitors, roflumilast is the first PDE4 inhibitor to be approved as a therapeutic drug to reduce the deterioration due to COPD [10]. However, as roflumilast has systemic side effects such as headache, weight loss, and vomiting, it is necessary to develop a therapeutic agent that can overcome these side effects [11].

Melatonin is a hormone secreted mainly from the pineal gland and has various pharmacological properties including anti-inflammatory, antioxidant, anti-apoptotic, and anti-tumor effects [12,13]. Melatonin is also generated in the peripheral organs, as well as the pineal gland, and produces its metabolites through a specific metabolism, which contributes to the maintenance of the proper function of the peripheral organs [14,15,16]. Due to the pharmacological properties of melatonin, protective and ameliorative effects by melatonin are observed in various respiratory diseases. In particular, we have demonstrated that melatonin alleviates lung inflammation induced by CS [17,18]. Melatonin reduced cigarette smoke condensate (CSC)-induced upregulation of mucin 5AC (MUC5AC) expression in human lung epithelial cells through suppression of mitogen-activated protein kinase signaling [17] and decreased the neutrophilic inflammatory response and mucin secretion induced by CS and lipopolysaccharide (LPS) through Erk-Sp1 signaling [18]. In addition, melatonin attenuated the pathophysiological condition of COPD via enhancement of SIRT1 expression and decreased the production of interleukin (IL)-8 induced by CS in pulmonary fibrosis [19,20,21]. Moreover, in clinical trials, melatonin reduced lung oxidative stress in patients with COPD [22]. However, the potential of melatonin as a PDE4 inhibitor in the treatment of COPD has not been examined.

We investigated the potential of melatonin as a PDE4 inhibitor in the treatment of COPD by exposing mice to CS and stimulating airway epithelial cells by cigarette smoke condensate (CSC). In addition, we studied the synergistic effects of melatonin and roflumilast, which is recommended as a PDE4 inhibitor in the treatment of COPD.

## 2. Results

### 2.1. Effect of Melatonin on Inflammatory Mediators in Mice Exposed to CS

**Figure 1:**
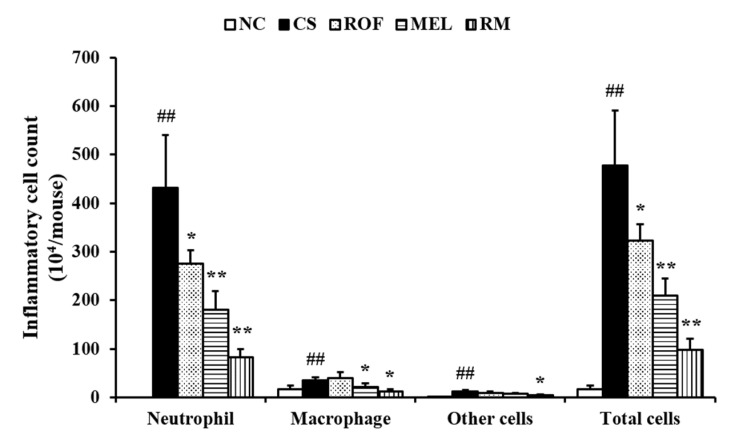
Melatonin decreased inflammatory cell counts in BALF of CS-exposed mice. Cells was determined by Diff-Quik^®^ staining and counted using a light microscope. NC: non-treated and non-exposure group; CS: PBS-treated and CS+LPS exposure group; ROF: roflumilast-treated and CS+LPS exposure group, MEL: melatonin-treated and CS+LPS exposure group, RM: roflumilast and melatonin co-treated and CS+LPS exposure group. The data are expressed as mean ± SD (n = 5). ^##^ Significantly different from the NC group, *p* < 0.01; *,** significantly different from the CS group, *p* < 0.05 or < 0.01, respectively.

Inflammatory cell count in the BALF of the CS group was higher than that in the BALF of the NC (Figure 1). However, the mice from the ROF and MEL groups exhibited significant reduction in inflammatory cell counts in BALF compared to those in the CS group. In particular, the counts were markedly reduced in the RM group (roflumilast and melatonin cotreatment) compared to those in the ROF or MEL groups.

**Figure 2:**
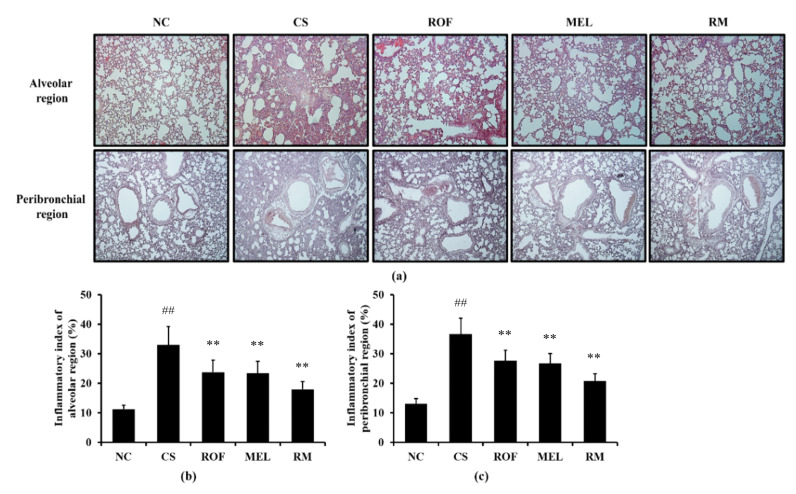
Melatonin reduced inflammatory cell infiltration in lung tissue of CS-exposed mice. The inflammatory cells infiltration was determined by H&E staining (n = 5). (**a**) lung histology stained with H&E, (**b**) inflammatory index of alveolar region, (**c**) inflammatory index of peribronchial region. NC: non-treated and non-exposure group; CS: PBS-treated and CS+LPS exposure group; ROF: roflumilast-treated and CS+LPS exposure group, MEL: melatonin-treated and CS+LPS exposure group, RM: roflumilast and melatonin co-treated and CS+LPS exposure group. The data are expressed as mean ± SD (n = 5). ^##^ Significantly different from the NC group, *p* < 0.01; ** significantly different from the CS group, *p* < 0.01.

Histological examination of the lung tissue revealed extensive inflammatory infiltration into the lung tissue of mice from the CS group (Figure 2). By contrast, ROF and MEL treatments decreased the pulmonary inflammation induced by exposure to CS. In addition, reduction in pulmonary inflammation was reduced to a greater extent in the RM group than in the ROF or MEL groups.

**Figure 3:**
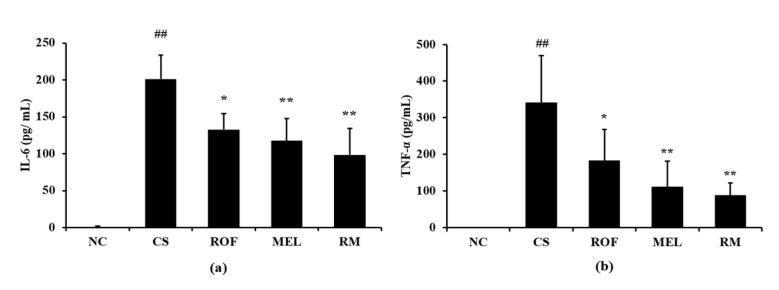
Melatonin decreased inflammatory cytokines in BALF of CS-exposed mice. The level of inflammatory cytokines was determined by ELISA assay. (**a**) Level of IL-6 and (**b**) level of TNF-α. NC: non-treated and non-exposure group; CS: PBS-treated and CS+LPS exposure group; ROF: roflumilast-treated and CS+LPS exposure group, MEL: melatonin-treated and CS+LPS exposure group, RM: roflumilast and melatonin co-treated and CS+LPS exposure group. The data are expressed as mean ± SD (n = 5). ^##^ Significantly different from the NC group, *p* < 0.01; *, ** significantly different from the CS group, *p* < 0.05 and *p* < 0.01, respectively.

IL-6 levels in BALF were higher in samples from the CS group than the NC group (Figure 3a). In contrast, ROF and MEL groups showed significantly decreased levels of IL-6 in BALF, compared to the CS group. The reduction in IL-6 levels was the most noticeable in the RM group. Similar to the IL-6 levels, the TNF-α levels in BALF were higher in the CS group than in the NC group (Figure 3b). However, the ROF, MEL, and RM groups showed significantly lower TNF-α levels in BALF than the CS group, with the most remarkable decrease in the RM group.

### 2.2. Effects of Melatonin on MMP-9, PDE4, and cAMP Levels in Mice Exposed to CS

**Figure 4:**
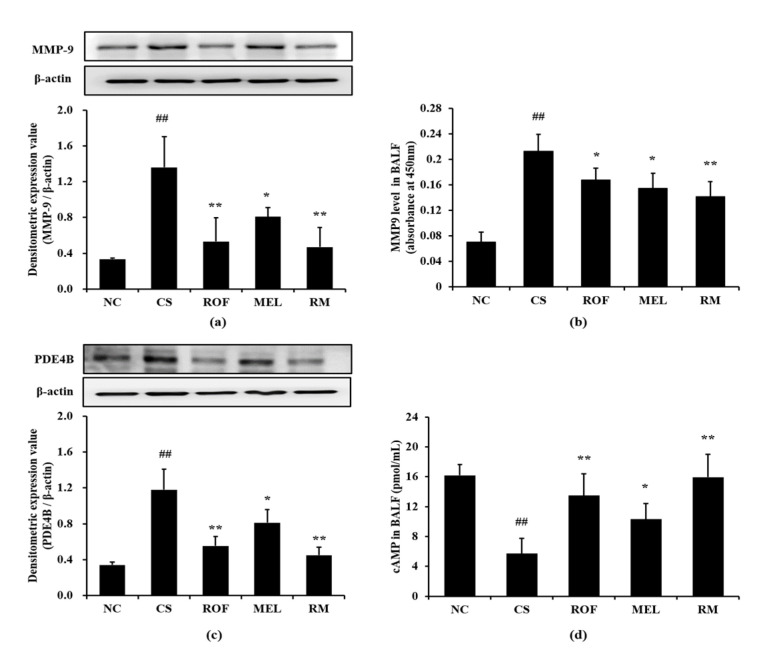
Melatonin reduced the elevation of MMP-9, PDE4B and cAMP in CS-exposed mice. (**a**) MMP-9 expression, (**b**) MMP-9 level in BALF, (**c**) PDE4B expression, (**d**) cAMP level in BALF. NC: non-treated and non-exposure group; CS: PBS-treated and CS+LPS exposure group; ROF: roflumilast-treated and CS+LPS exposure group, MEL: melatonin-treated and CS+LPS exposure group, RM: roflumilast and melatonin co-treated and CS+LPS exposure group. The data are expressed as mean ± SD (n = 5). ^##^ Significantly different from the NC group, *p* < 0.01; *, ** significantly different from the CS group, *p* < 0.05 and < 0.01, respectively.

Mice in the CS group showed marked elevation in MMP-9 expression in lung tissues compared to mice from the NC group (Figure 4a). In contrast, the ROF, MEL, and RM groups showed a significant decrease in MMP-9 expression, with the RM group showing the most noticeable reduction. Further, the MMP-9 level was markedly increased in BALF in the CS group compared to the NC group (Figure 4b). However, ROF and MEL groups showed significantly lower MMP-9 level than the CS group. This reduction was more remarkable in the RM group. Consistently, PDE4B expression was markedly increased in the CS group compared to the NC group; however, the ROF and MEL groups showed significantly decreased PDE4B expression, compared to the CS group (Figure 4c). PDE4B expression was reduced to a much greater extent in the RM group than in the ROF or MEL groups. The cAMP level in BALF was markedly decreased in the CS group, compared to the NC group; however, it was significantly increased in ROF, MEL, and RM groups compared to the CS group (Figure 4d), with the RM group showing the greatest degree of elevation.

### 2.3. Effects of Melatonin on Lung Function in Mice Exposed to CS

**Figure 5:**
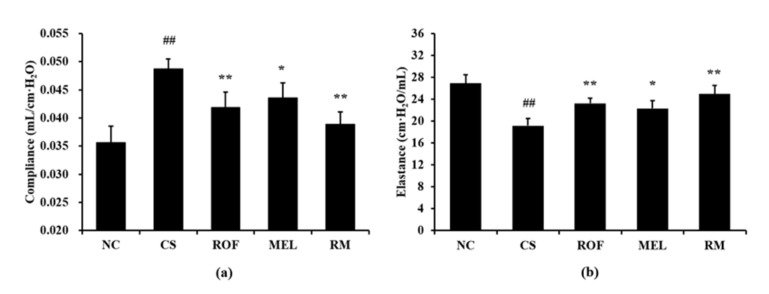
Melatonin restored worsened lung function in CS-exposed mice. (**a**) Compliance, (**b**) elastance. NC: non-treated and non-exposure group; CS: PBS-treated and CS+LPS exposure group; ROF: roflumilast-treated and CS+LPS exposure group, MEL: melatonin-treated and CS+LPS exposure group, RM: roflumilast and melatonin co-treated and CS+LPS exposure group. The data are expressed as mean ± SD (n = 5). ^##^ Significantly different from the NC group, *p* < 0.01; *, ** significantly different from the CS group, *p* < 0.05 and < 0.01, respectively.

The CS group showed markedly increased lung compliance than the NC group (Figure 5a). However, compared to the CS group, the ROF and MEL groups exhibited significant reduction in lung compliance. In particular, lung compliance was reduced to a greater extent in the RM group than in the ROF or MEL group. The CS group showed a significant reduction in elastance compared to the NC group (Figure 5b). In contrast, the ROF, MEL, and RM groups exhibited a marked elevation of elastance compared to the CS group, with the RM group showing the greatest degree of elevation.

### 2.4. Effect of Melatonin on Inflammatory Mediators in CSC Stimulated NCI-H292 Cells

**Figure 6:**
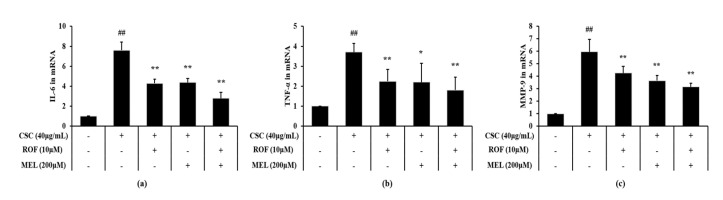
Melatonin reduced mRNA expression of inflammatory cytokines and MMP-9 in CSC-stimulated cells. The level of mRNA expression of inflammatory cytokines and MMP-9 was determined by real-time PCR. (**a**) *IL-6*, (**b**) *TNF-α,* (**c**) *MMP-9*. This experiment was repeatedly performed three times. ^##^ Significantly different from the non-treated cells, *p* < 0.01; *,** significantly different from the CSC-stimulated cells, *p* < 0.05 and < 0.01, respectively.

Expression levels of IL-6 and TNF-α were significantly increased in CSC-stimulated cells compared to non-treated cells (Figure 6a,b, respectively). However, melatonin treatment significantly decreased the IL-6 and TNF-α expression levels in CSC-stimulated cells. Roflumilast treatment also yielded similar results. Furthermore, roflumilast and melatonin cotreatment decreased the IL-6 and TNF-α expression levels to a greater extent than roflumilast or melatonin treatment alone. Consistently, MMP-9 expression was higher in CSC-stimulated cells than in the non-treated cells (Figure 6c). By contrast, melatonin treatment significantly reduced MMP-9 expression, compared with CSC-stimulated cells. Similar results were obtained with roflumilast treatment. Moreover, this reduction was more enhanced in roflumilast and melatonin cotreated cells.

### 2.5. Effect of Melatonin on PDE4B Expression and cAMP Activity in CSC-Stimulated NCI-H292 Cells

**Figure 7:**
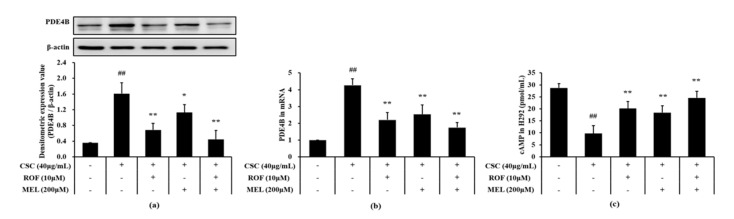
Melatonin decreased PDE4B and cAMP in CSC-stimulated cells. The protein and mRNA expression of PDE4B was determined in by immunoblotting and real-time PCR, respectively. cAMP level was determined in supernatant by ELISA. (**a**) PDE4B expression, (**b**) mRNA expression of *PDE4B*, (**c**) level of cAMP. This experiment was repeatedly performed three times. ^##^ Significantly different from the non-treated cells, *p* < 0.01; *,** significantly different from the CSC-stimulated cells, *p* < 0.05 and *p* < 0.01.

Compared to non-treated cells, CSC-stimulated cells exhibited a significant increase in the PDE4B protein and mRNA expression levels (Figure 7a,b, respectively). However, melatonin treatment significantly suppressed the elevation of the PDE4B protein and mRNA expression in CSC-stimulated cells. Furthermore, roflumilast and melatonin cotreatment decreases the PDE4B protein and mRNA expression levels to a greater extent than roflumilast or melatonin treatment alone. Compared to the non-treated cells, the cAMP level was markedly reduced in CSC-stimulated cells (Figure 7c). However, melatonin treatment resulted in a significant increase in cAMP level, compared to CSC-stimulated cells. Similar results were obtained with roflumilast treatment. In addition, roflumilast and melatonin cotreatment resulted in the increase in cAMP levels; this increase was more than that obtained with roflumilast or melatonin treatment alone.

## 3. Discussion

Melatonin is used for the treatment of various diseases due to its pharmacological properties [17,18]. Although our previous study has shown that melatonin is effective in treating COPD, the exact mechanism of action of melatonin in the treatment of COPD is still very poorly understood [18]. In this study, we investigated the potential of melatonin to serve as a PDE4 inhibitor in the treatment of COPD using mice exposed to CS and CSC-stimulated cells. Melatonin effectively suppressed inflammatory cell infiltration, production of proinflammatory cytokines and expression of MMP-9 in mice exposed to CS and CSC-stimulated cells, which was accompanied with a reduction in PDE4 expression and elevation of cAMP levels. Due to these responses, melatonin ameliorated the CS-exposure-induced worsening of lung function. In addition, these responses were further enhanced upon cotreatment with melatonin and roflumilast.

CS is considered a crucial contributor in the development of COPD [23,24,25,26]. Exposure to CS stimulated the recruitment of many inflammatory cells such as neutrophils and macrophages into lung tissues; these cells produce various inflammatory mediators including cytokines, reactive oxygen species, chemokines, and MMPs [25,26,27,28]. Because of these responses, the normal structure of the lung tissue is destroyed, resulting in declined lung function [26,27,28]. In our study, melatonin treatment inhibited the elevation of inflammatory cell count, cytokine levels, and MMP-9 expression in mice exposed to CS, with a reduction in the levels of inflammatory mediators in CSC-stimulated cells. Consistently, administration of melatonin effectively suppressed the recruitment of inflammatory cells into the lung tissues in mice exposed to CS. In particular, these responses were found to be pronounced upon cotreatment with melatonin and roflumilast. These results indicate that melatonin can effectively inhibit the development of COPD, and that cotreatment with roflumilast has a better therapeutic effect than the administration of either agent alone.

PDE4 inhibitors are clinically recommended drugs for the treatment of COPD [29]. PDE4 inhibitors inhibit the conversion of cAMP to AMP, thereby reducing inflammatory responses in the lung tissue and normalizing lung function [30,31]. During the development of COPD, exposure to CS increases the expression of PDE4 in the lung tissue, thereby reducing the levels of cAMP. This eventually worsens inflammatory responses in the lung tissue and causes a decrease in lung function [32,33]. Thus, PDE4 inhibitors are recognized as very important drugs in the treatment of COPD, and many researchers are currently focused on discovering COPD drugs, with an emphasis on PDE4 inhibition of candidate drugs [34,35,36,37]. In previous studies, PDE4 inhibitors reduced lung inflammation and restored lung function by increasing cAMP through inhibition of the PDE4 expression in CS-induced COPD animal models. In this study, administration of melatonin effectively reduced the expression of PDE4 caused upon CS exposure, increased the levels of cAMP, and led to the recovery of lung function following CS-exposure-induced decline in lung function. These responses were more noticeably detected upon cotreatment with melatonin and roflumilast. In addition, the same was observed in CSC-stimulated cells. Treatment with melatonin suppressed PDE4 expression in CSC-stimulated cells, with an elevation in the cAMP levels. This result indicated that the therapeutic effect of melatonin in COPD is associated with the suppression of PDE4, and when combined with the PDE4 inhibitor, its therapeutic effect in COPD is greatly elevated.

The synergistic effects of melatonin and roflumilast against COPD is considered to be related with its PDE4B inhibitory effect and other pharmacological properties, such as anti-inflammatory and antioxidative effects. There have been various experiments related to the anti-COPD effect of melatonin [18,19,20,21,22,38,39]. In previous studies, the anti-COPD effect of melatonin was associated with the anti-inflammatory and antioxidant effects of melatonin in in vivo and in vitro experiments using CS or CSC [18,19,20,21,38,39]. In this study, it is suggested that the anti-COPD effect of melatonin may be related not only to its anti-inflammatory and antioxidant properties, but also to the ability of inhibiting PDE4B expression. Therefore, it is considered to exhibit a synergistic effect when administered in combination with roflumilast due to the inhibitory effect of PDE4B expression and other pharmacological properties of melatonin.

In summary, treatment of melatonin attenuates inflammatory responses in CS-exposed mice and CSC-stimulated cells, which is correlated with elevation of cAMP via inhibition of PDE4 expression. Our results suggest that melatonin has potential as a PDE4 inhibitor in the treatment of COPD.

## 4. Materials and Methods

### 4.1. Cell Culture and Viability Assay

The NCI-H292 cell line was obtained from the ATCC (Manassas, VA, USA). Cells were cultured in RPMI 1640 (WELGENE, Gyeongsangbuk-do, Republic of Korea) supplemented with 10% heat-inactivated fetal bovine serum and antibiotics (WELGENE). The viability assay was performed using 3-(4,5-dimethylthiazol-2-yl)-2,5-diphenyltetrazolium bromide (MTT, Sigma-Aldrich, Saint Louis, MO, USA). Cells were maintained in 96-well plates at a density of 3 × 10^4^ cells/well. Roflumilast (ROF, Sigma-Aldrich) and melatonin (MEL, Sigma-Aldrich) were added to each well as follows: ROF: 2.5, 5, 10, and 20 μM; MEL: 100, 200, 400, and 800 μM. This was followed by incubation for 24 h. MTT solution (10 μL) was added to each well, and the cells were incubated for 4 h at 37 °C; then, 100 μL of dimethyl sulfoxide (DMSO, Sigma-Aldrich) was added to each well to solubilize the formazan produced. The optical density of the culture was measured at 570 nm.

### 4.2. RNA Isolation and Real-Time PCR

NCI-H292 cells were seeded on 60 mm dishes at a density of 1 × 10^6^ cells/well and were treated with ROF (10 μM) and MEL (200 μM), for 1 hour. After incubation, cells were stimulated with CSC. CSC was prepared as previously described [18]. Total RNA was isolated using RNA isolation kit (Invitrogen, Carlsbad, CA, USA). cDNA was synthesized using Oligo DTs and the cDNA synthesis kit (Qiagen, Hilden, Germany). Polymerase chain reactions were performed using specific forward and reverse primers (TNF-α, forward, 5ʹ-CAAAGTAGACCTGCCCAGAC-3ʹ, reverse, 5′- GACCTCTCTCTAATCAGCCC-3ʹ; IL-6, forward, 5ʹ-ATGCAATAACCACCCCTGAC-3‘ and reverse, 5ʹ- ATCTGAGGTGCCCATGCTAC-3ʹ; MMP-9, forward, 5ʹ-AAGGGCGTCGTGGTTCCAACTC-3ʹ and reverse, 5ʹ-AGCATTGCCGTCCTGGGTGTAG-3ʹ; PDE4B, forward, 5ʹ-ATTGTAGCAATGGACAGAC-3ʹ and reverse, 5ʹ-GTATCGAGATCCTGAGCATC-3ʹ; GAPDH, forward, 5ʹ- CAAAAG GGTCATCTCTG-3ʹ, reverse, 5ʹ- CCTGCTTCACCACCTTCTTG-3ʹ). The mRNA expression levels of the target genes were normalized to that of housekeeping gene GAPDH.

### 4.3. CS Induced Airway Inflammation

Male C57BL/6 mice (6–8 weeks old, 20–25g) were purchased from Semtaco Co (Osan, Korea). Mice were housed in standard conditions. All procedures were approved by the Institutional Animal Care and Use Committee of the Chonnam National University (CNU IACUC-YB-R-2016-18). CS-induced airway inflammation model was established as explained in a previous study [40]. Briefly, mice were exposed to 3R4F research cigarettes (Kentucky reference cigarette, University of Kentucky, USA) for 14 days using a CS generator (Daehan Biolink, Inchun, Korea) and LPS (5 μg/mouse, Sigma-Aldrich) was administered intranasally on day 5. ROF (10 mg/kg) and MEL (30 mg/kg) were intraperitoneally administered 1 hour before exposure to CS for 14 days. Mice were randomly divided into five groups as follows (n = 5): NC (normal control; no treatment + fresh air and PBS exposure), CS (PBS treatment + CS and LPS exposure), ROF (roflumilast treatment + CS and LPS exposure), MEL (melatonin treatment + CS and LPS exposure), and RM (roflumilast and melatonin treatment + CS and LPS exposure). Lung function was evaluated using Flexivent (SCIREQ Inc., Montreal, QC, Canada) 24 h after the last CS exposure. The animals were subjected to tracheostomy, and detection probe was inserted into trachea. The compliance and elastance of each animal were evaluated according to the programmed protocols of the instrument.

### 4.4. Bronchoalveolar Lavage Fluid (BALF) Collection and Analysis

BALF collection was performed as described previously [12]. Animals were subjected to tracheostomy, and cold PBS (0.7 mL) was infused into the lung tissue and withdrawn via tracheal cannulation twice (total, 1.4 mL). The BALF thus obtained was centrifuged at a speed of 1500 rpm for 5 min, and then the supernatant was used to evaluate cytokine and cAMP activity. Cell pellets were used to determine inflammatory cell counts in the BALF. To evaluate differential cell count, cells were attached on slides and stained with Diff-Quik reagent (IMEB, Deerfield, IL, USA). Total cell count was determined using an automatic cell count analyzer (Thermo Fisher Scientific, San Diego, CA, USA). Levels of cytokines (R&D System, Minneapolis, MN, USA) and activity of cAMP (Abcam, Cambridge, UK) were evaluated using an ELISA kit according to the manufacturer’s instructions.

### 4.5. Immunoblotting

The lung tissue was homogenized (1/10 *w*/*v*) using a homogenizer in tissue lysis/extraction reagent (Sigma-Aldrich) supplemented with protease inhibitors (Sigma-Aldrich). Immunoblotting was performed as described previously [12]. The following primary antibodies were used: anti-PDE4B (1:1000 dilution; Abcam), anti-MMP-9 (1:1000 dilution; Abcam), and anti-β-actin (1:1000 dilution; Cell signaling, Denver, MA, USA). Expression of PDE4B, MMP-9, and β-actin were evaluated using ChemiDoc (Bio-Rad, Hercules, CA, USA).

### 4.6. Histological Analysis

The right lungs of mice from each group were fixed in 10% buffered formalin (Sigma-Aldrich) for 3 days at room temperature. Fixed lung tissue was embedded in paraffin blocks, cut into 4 μm thick sections, deparaffinized using xylene, and dehydrated using ethanol. Following a 5 min wash with distilled water, the tissue sections were stained using hematoxylin and eosin stain to evaluate inflammatory cell infiltration. Quantification of inflammatory responses was conducted using an image analyzer (IMT i-Solution Ins., Vancouver, BC, Canada).

### 4.7. Statistical Analysis

Data are presented as the means ± standard deviation (SD). Statistical significance of results was determined using ANOVA followed by the multiple comparison test with Dunnet’s adjustment and were considered significant at *p* < 0.05.

## Data Availability

Data is contained within the article.
